# Hot Plate Annealing at a Low Temperature of a Thin Ferroelectric P(VDF-TrFE) Film with an Improved Crystalline Structure for Sensors and Actuators

**DOI:** 10.3390/s141019115

**Published:** 2014-09-26

**Authors:** Rahman Ismael Mahdi, W.C. Gan, W. H. Abd. Majid

**Affiliations:** 1 Low Dimensional Materials Research Centre, Department of Physics, University of Malaya, Kuala Lumpur 50603, Malaysia; E-Mail: wcgan1981@gmail.com; 2 Nanotechnology and Advanced Materials Research Centre, University of Technology, Baghdad 10066, Iraq

**Keywords:** ferroelectric copolymers, crystallinity, dielectric, pyroelectric

## Abstract

Ferroelectric poly(vinylidene fluoride-trifluoroethylene) (P(VDF-TrFE)) copolymer 70/30 thin films are prepared by spin coating. The crystalline structure of these films is investigated by varying the annealing temperature from the ferroelectric phase to the paraelectric phase. A hot plate was used to produce a direct and an efficient annealing effect on the thin film. The dielectric, ferroelectric and pyroelectric properties of the P(VDF-TrFE) thin films are measured as a function of different annealing temperatures (80 to 140 °C). It was found that an annealing temperature of 100 °C (slightly above the Curie temperature, Tc) has induced a highly crystalline β phase with a rod-like crystal structure, as examined by X-ray. Such a crystal structure yields a high remanent polarization, *P*_r_ = 94 mC/m^2^, and pyroelectric constant, *p* = 24 μC/m^2^K. A higher annealing temperature exhibits an elongated needle-like crystal domain, resulting in a decrease in the crystalline structure and the functional electrical properties. This study revealed that highly crystalline P(VDF-TrFE) thin films could be induced at 100 °C by annealing the thin film with a simple and cheap method.

## 1. Introduction

Piezo-, pyro- and ferroelectric polymers receive a lot of attention, because of their potential applications in sensors and actuators [1]. These electrical properties can only be found in non-centrosymmetric materials. In order to break the centrosymmetry, the polymers usually have to be polarized. As a result of the polarization process, the domains and/or dipoles orientate and layers of charges are built-up in the heterogeneous polymer materials. Both effects can also occur simultaneously. Poly(vinylidene fluoride), PVDF, and its copolymers are strongly investigated due to their outstanding piezo-, pyro- and ferroelectric properties compared to other polymers. These properties lead to the development of interesting technological applications, like sensors and actuators [2,3]. PVDF exhibits several different crystalline phases (called α,β,γ,δ) and morphologies depending on the processing conditions [1,4]. It is a high molecular weight (M_w_ ≈ 106 g·mol^−1^) semi-crystalline polymer with a degree of crystallinity that can range from 45% to 60% [1,3]. PVDF is a linear polymer that shows permanent electric dipoles approximately perpendicular to the direction of the chains. These dipoles are due to the difference in electronegativity between the atoms of hydrogen and fluorine with respect to carbon [5–8]. The glass transition temperature T_g_ for PVDF is around −34 °C, and the melting temperature, T_m_, ranges between 165 and 189 °C, depending on the crystalline phase present in the polymer [9]. The piezoelectric properties of the material are related to the crystalline phase, orientation and polarization state of the polymer. The β phase of PVDF is the phase that exhibits the best ferroelectric and piezoelectric properties.

The copolymer crystal structure, phase transition behavior and ferroelectric properties are affected by the ratio of VDF/fluoride-trifluoroethylene (TrFE) content and the synthesizing conditions [10]. Similar to PVDF, P(VDF-TrFE) has been reported to crystallize into four types of crystalline phases (α,β,γ,δ). P(VDF-TrFE) can easily form the β phase by adding a small amount of TrFE. The copolymer exhibits a much higher crystalline phase than PVDF does. P(VDF-TrFE) can also be crystallized into the β phase by heat treatment at a temperature between the Curie transition temperature (T_c_) and the melting temperature (T_m_) [9]. Heat treatment is an essential and important procedure in fabricating thin films. Annealing can be used to improve the crystalline structure, reduce the porosity and ensure the elimination of the residual solvent used during the fabrication process of P(VDF-TrFE) thin films. The temperature used for annealing, the time of annealing and the rate of ramping up and cooling are three important parameters in the thermal annealing process [11]. Annealing temperature must be above the Curie temperature, T_c_, and below the melting point, T_m_, of the material when the material is in between the ferroelectric phase and the paraelectric phase. The thermal energy allows the polymer chains to rearrange their orientation and position, such that a higher degree of crystalline structure can be formed after cooling [9].

In view of current economic solicitude, as well as technical compatibility with thermal treatment, low temperature processing is a critical issue to realize the large-area integration of active layers with flexible electronics. This platform is not only important for organic materials, but also applicable for many other functional oxide materials [12–14]. In this study, we have focused on improving the crystalline structure of P(VDF-TrFE) 70/30 by annealing the samples with a hot plate. In this technique, the heat came only from one direction, *i.e.*, from the bottom of a substrate. The fluctuation in the temperature was reduced to ±3 °C. A high crystalline structure can be achieved by using this technique at 100 °C. In the previously reported studies, the lowest heating temperature needed to optimize the crystalline structure was around 140 °C [1,9,11]. If the temperature is closer to the melting point of the copolymer, it will be partially melted, and that will have an effect on the degree of the crystalline structure. The fluctuation in the temperature inside an oven is around ±15 °C, as the heat transferred through air inside an oven comes from many different directions.

## 2. Experimental Details

P(VDF-TrFE) 70/30 powder was supplied by Kureha, Japan. The powder was dissolved with methyl ethyl ketone solvent (MEK) in 5 wt%. The solution was stirred for three hours with a magnetic stirrer at 120 °C. This step was taken to ensure the complete dissolution of P(VDF-TrFE) powder. An aluminum electrode was deposited on a clean glass substrate. The films were then spun on an Al electrode and silicon substrates at 8,000 rpm. Prior to the deposition of the top Al electrodes being performed, the films were put in an oven to dry for 4 h at 60 °C. In order to enhance the crystalline structures, the thin films were annealed for 60 min at several different temperatures (80, 90, 100, 110, 120 and 140 °C). We used a hot plate to anneal the thin film, such that the substrate was in direct contact with the hot plate surface. The technique helped to reduce the fluctuation of the annealing temperature, rather than using an oven, where the heat transfer in the oven by convection usually has an error of ±15 °C. The temperature was carefully measured on the top of the hot plate and on the top of the thin films. The temperature difference in between both methods was found to be around ±3 °C. Finally, the top electrode was deposited to form a sandwich structure. The thickness of the thin films as measured by a KLA-Tencor P-6 profilometer is 220 nm. The Curie temperature of such films was 90 °C, and the melting temperature was above 140 °C, as determined from the phase transition [9]. The effective electrode area used was 1 mm^2^ for dielectric and pyroelectric measurements. The effective electrode area for ferroelectric measurement was 0.15 mm^2^. The lower effective electrode area is used in the ferroelectric measurement to prevent dielectric breakdown and arcing in the active area when a high electric field was applied. The ferroelectric hysteresis loop was obtained using a Precision LC Analyzer. The capacitance bridge method with high accuracy and a wide frequency range was used to investigate the dielectric properties of the thin films. The measurement of the real (ε′) and the imaginary (ε″) part of the complex dielectric permittivity, ε*, is represented by ε* = *ε*′ − *iε*″.

The measurements were carried out from room temperature (27 °C) to 140 °C with an impedance analyzer, Agilent 4294A (40 Hz–110 MHz). A quasi-static method was employed to measure the pyroelectric coefficient of the thin films. The temperature of the sample was increased and decreased at the constant rate of the non-radiative heat source, which generates the triangular temperature waveform, while the short-circuited pyroelectric current was measured. The triangular temperature was generated using a Lakeshore temperature controller. The pyroelectric current *I*_p_, which was generated when the P(VDF-TrFE) thin film was heated and cooled repeatedly, was measured by a Keithely 617 electrometer. The crystalline phase of the samples was examined by an X-ray diffractometer (XRD) PANalytical-Empyrean, and a Perkin Elmer 2000 Fourier Transform Infrared (FTIR) spectroscopy system. A field electron scanning electron microscope (FESEM) was obtained with a Hitachi (SU8000).

## 3. Results and Discussion

### 3.1. Structural Analysis

**Figure 1.**
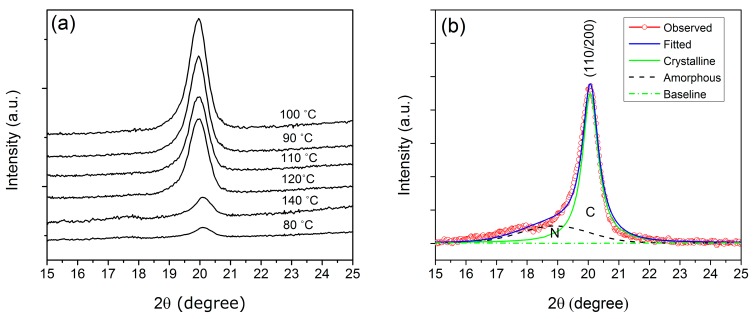
(**a**) XRD result of poly(vinylidene fluoride-trifluoroethylene) (P(VDF-TrFE)) thin films of different annealing temperatures (80, 90, 100, 110, 120, 140 °C); (**b**) Deconvolution of the X-ray diagram for P(VDF-TrFE) thin film annealed at 100 °C.

The crystal structure of P(VDF-TrFE) is normally related to the composition (mole ratio of P(VDF/TrFE) of the copolymer and the annealing process. In the β crystalline phase of P(VDF-TrFE), the unit cell is orthorhombic, with each chain aligned and packed with the CF_2_ groups parallel to the b-axis [11,15]. Figure 1a shows the XRD pattern of P(VDF-TrFE) for different annealing temperatures (80, 90, 100, 110, 120, 140 °C) to obtain information on the degree of the crystalline structure of the copolymer thin films. Characteristic peaks, associated with the β phase, appearing at 2θ = 19.90°, are assigned to (110/200) reflection planes. An elevated diffraction peak indicates a high percentage of the crystalline structure in the β phase. One can note from the XRD result that the peak intensity at 80 °C was one of the lowest, because the annealing temperature was below the Curie temperature of the materials, which is not enough to align the chains. However, the highest intensity peak was achieved at 100 °C, which means that the crystalline structure of the copolymer thin film is the highest. The Curie temperature of P(VDF-TrFE) 70/30 is around 90 °C, where the β phase starts to change above 100 °C. The crystalline structure of the thin film obtained when annealed around 100 °C will be more favorable for the ferroelectric, pyroelectric and dielectric properties. This is due to the high crystalline structure exhibited by the composite thin film, which will be discussed in the following sections. One can see from Figure 1a that the intensity decreases at annealing temperatures of 120 °C and 140 °C dramatically. As the materials were in the paraelectric phase, they have lost their ferroelectric properties. In this condition, the molecules and atoms move randomly, which could lead to loose crystalline structure, as has been observed in the XRD result.

**Figure 2.**
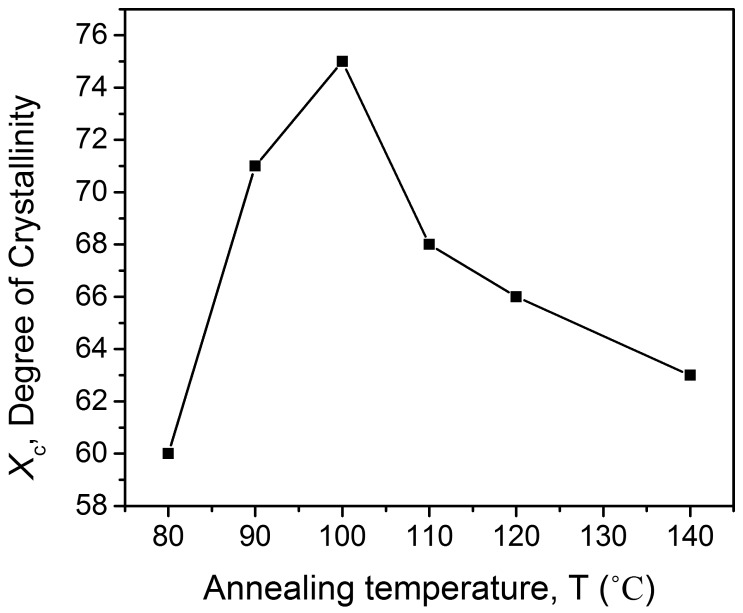
Dependence of the degree of the crystalline structure as a function of annealing temperature.

The observed diffraction curves could be resolved into two peaks, C (crystalline) and N (non-crystalline), as shown in Figure 1b. The deconvolution was performed by fitting to a superposition of a Gaussian function to determine the integrated peak areas and refinement of the peak positions. The peaks in this region are analogous to the β phase crystalline plane, whereas the shoulder is associated with the halo from the noncrystalline molecules. As a result, the diffraction curve observed was resolved into two regions, crystalline (green solid lines) and amorphous (black dashed line). The degree of the crystalline structure, *X*_c_, was evaluated from the ratio of area C to the total area under the diffraction curves N + C. The obtained *X*_c_ was 75%, and it is clearly seen in Figure 2 that annealing at 100 °C induces a great increase in *X*_c_.

### 3.2. Surface Morphology

**Figure 3.**
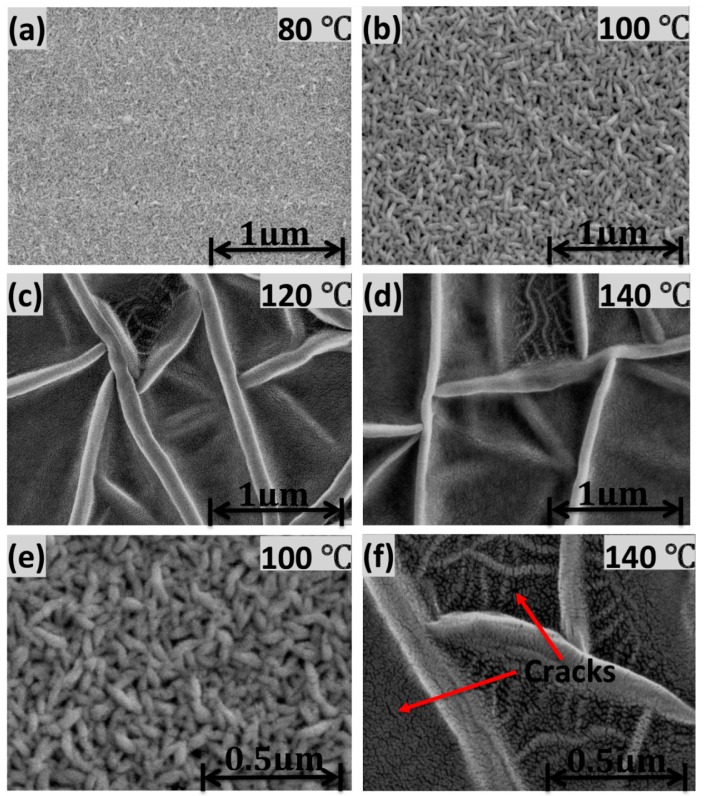
FESEM images of P(VDF-TrFE) annealed at (**a**) 80 °C; (**b**) 100 °C; (**c**) 120 °C; (**d**) 140 °C; (**e**) An amplified picture of (b); (**f**) an amplified picture of (d).

Figure 3 shows the surface morphology of the annealed P(VDF-TrFE) thin films as observed using FESEM at 50K magnification. The 80 °C annealed thin films produced the morphology of the undefined crystalline structure. Significant elongated crystalline structures of 90 nm in length were clearly observed when the annealing temperature was increased to 100 °C. However, when the annealing temperature was increased to 120 °C, the length of the elongated crystalline increased to 5.5 μm long. The length of the elongated crystalline continued to increase to 15.5 μm when the copolymer was annealed at 140 °C. In general, P(VDF-TrFE) thin films are annealed between the Curie and melting temperatures in order to induce the crystalline structure. In the paraelectric phase, the chain mobility is higher compared to that of the ferroelectric phase. It favors the lowest energy conformation (all *trans*), because as the temperature increases, the chain mobility increases as a function of temperature. As a result, the molecular chains prefer being oriented in parallel to the substrate, and rod-like crystals are observed when the annealing temperature reached 100 °C, as shown in Figure 3b. This suggests that small crystallites undergo a transition into a paraelectric phase. They grew by incorporating surrounding non-crystalline molecules and thus contributed to an increase in the crystalline structure. On the other hand, annealing above 100 °C resulted in the formation of acicular grains (needle-like crystals) in edge-on lamellae, as shown in Figure 3c,d. Near the melting point, the morphology changes drastically, due to the small crystals being partially fused and recrystallized. In such a way, the chain axis is reoriented normal to the substrate surface. This implies that annealing at 100 °C induced growth in the crystalline structure as a result of the coalescence of neighboring crystallites, as indicated in Figure 2. The significant change in the morphology of the surface for the thin films annealed above 100 °C, can be clearly observed with the combination of some defects, such as a crack in the surface, as shown in Figure 3f, an amplified picture of Figure 3d. The defects will lead to a decrease in the electrical properties of the material, as explained in the following analysis.

### 3.3. Vibrational Analysis for β Phase Dominant Crystals

**Figure 4.**
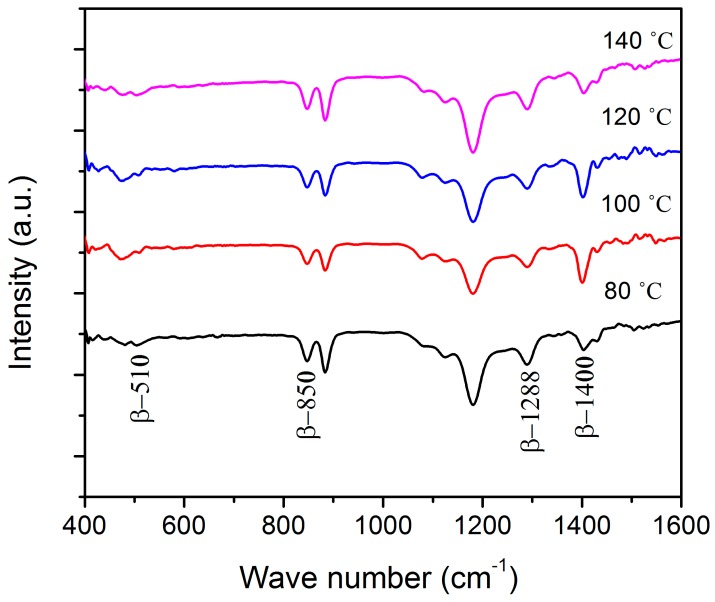
FTIR spectra of P(VDF-TrFE) thin films treated at different annealing temperatures.

The incident electromagnetic field from the IR source interacts with the molecular bonding of the P(VDF-TrFE) film. The interaction has resulted in a large absorption when the molecular vibration and the electric field component of the IR are perpendicular to each other. Each phase of the P(VDF-TrFE) copolymer will provide a characteristic FTIR spectrum.Due to the large mass of the fluorine atom, most infrared-active vibrations for the copolymer are concentrated in a rather narrow region, 400–1500 cm^−1^ [16]. Details of the absorption band assignments can be found in the literature [11,16–18]. Figure 4 illustrates the transmission infrared (IR) spectra of P(VDF-TrFE) thin films annealed at different temperatures. Four intense bands were discussed, which are 510 cm^−1^, 850 cm^−1^, 1288 cm^−1^ and 1400 cm^−1^, which are due to the β phase of P(VDF-TrFE). The 510 cm^−1^ and 1400 cm^−1^ bands are assigned to the CF_2_ bending mode within *TTT* segments of the chain and the wagging vibration of CH_2_, respectively [16,17]; whilst the bands at 850 cm^−1^ and 1288 cm^−1^ are assigned to CF_2_ symmetric stretching mode [16]. The intensity and the position of the absorption bands do not change much when treated with different annealing temperature within the paraelectric phase. This implied that an annealing temperature below the melting point did not affect the structure and crystalline phase of the copolymer in the β phase.

### 3.4. Ferroelectric Properties

**Figure 5.**
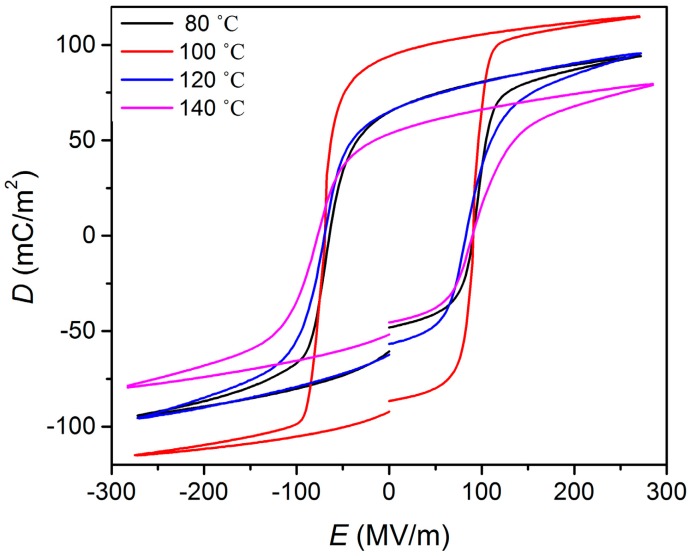
*D-E* hysteresis loops of P(VDF-TrFE) as a function of annealing temperature.

**Figure 6.**
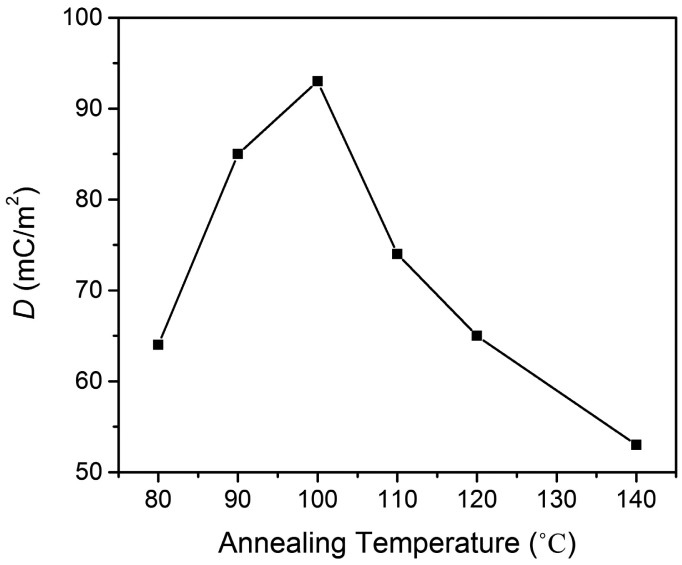
Dependence of remnant polarization, *P*_r_, as a function of annealing temperature.

The hysteresis loop is important in characterizing ferroelectric properties. A significant amount of information can be extracted from the hysteresis loop. For the investigation of the influence of an external electric field on the dipole moment and polarization of P(VDF-TrFE), the direction of the applied external electric field *E* is along the total average dipole moment of the molecular unit. Its rotation to the opposite one leads to a switching of the molecular dipole. This occurs when the electric field exceeds the critical coercive field *E*_c_ [19]. The driving force for the reorientation is the minimization of the total energy of the modeled molecular system, which changes in different dipole configurations. *D-E* hysteresis loops of P(VDF-TrFE) as a function of annealing temperature are presented in Figure 5. The hysteresis measurements were obtained at 100 Hz with different applied voltages for P(VDF-TrFE) thin films of a thickness of approximately 220 nm at room temperature. The highest remnant polarization of *P_r_* = 94 mC/m^2^ with a *E*_c_ = 80 MV/m was obtained from the thin films annealed at 100 °C. As discussed previously in the XRD section, the thin film annealed at 100 °C has the highest crystalline structure. The shape of the optimized hysteresis loop of the thin film annealed at 100 °C is almost a square, which indicates the excellent ferroelectric properties of the thin films. Compare our value of *P*_r_ = 94 mC/m^2^ to the previous reported research, for example D. Mao *et al.* [20]: the *P*_r_ obtained for the thin film annealed at 140 °C by an oven is 82 mC/m^2^. As we have used a hot plate for annealing the thin film, the distribution of thermal energy to the thin film is expected to be more homogeneous than that of an oven, so a high degree of crystalline chains and, hence, high spontaneous polarization can be achieved. The *P*_r_ for the thin film annealed at the other annealing temperature decreased considerably to less than 60 mC/m^2^, as shown in Figure 6.

### 3.5. Dielectric Analysis

**Figure 7.**
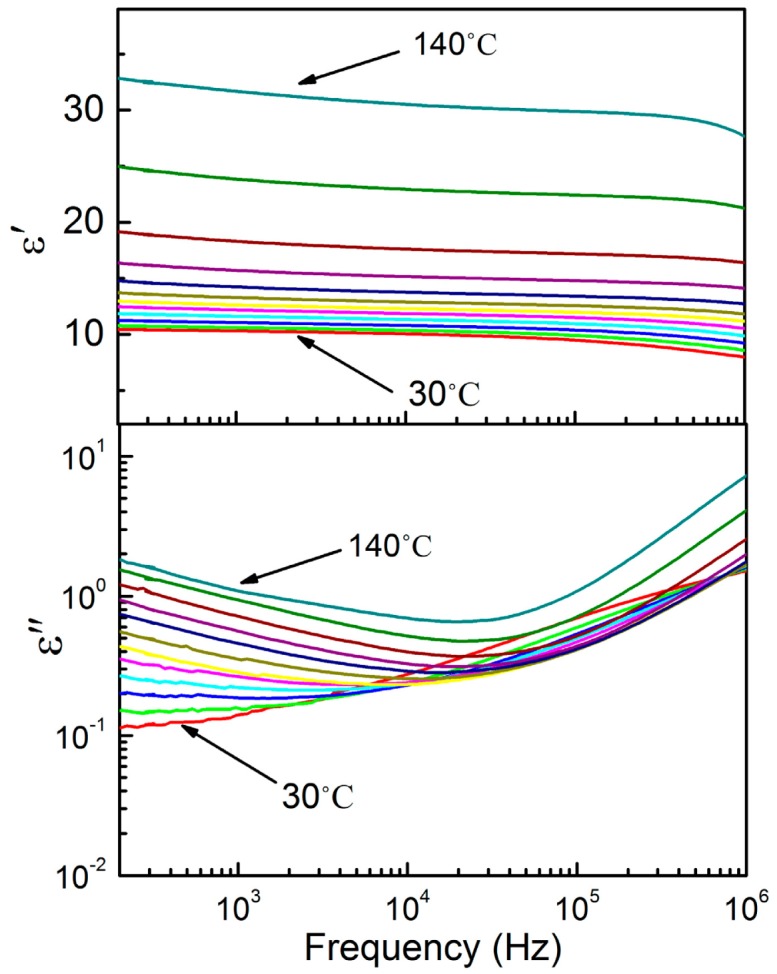
Dielectric frequency spectra for the P(VDF-TrFE) thin film annealed at 100 °C and measured at a temperature step size of 10 °C from 30 °C to 140 °C.

**Figure 8.**
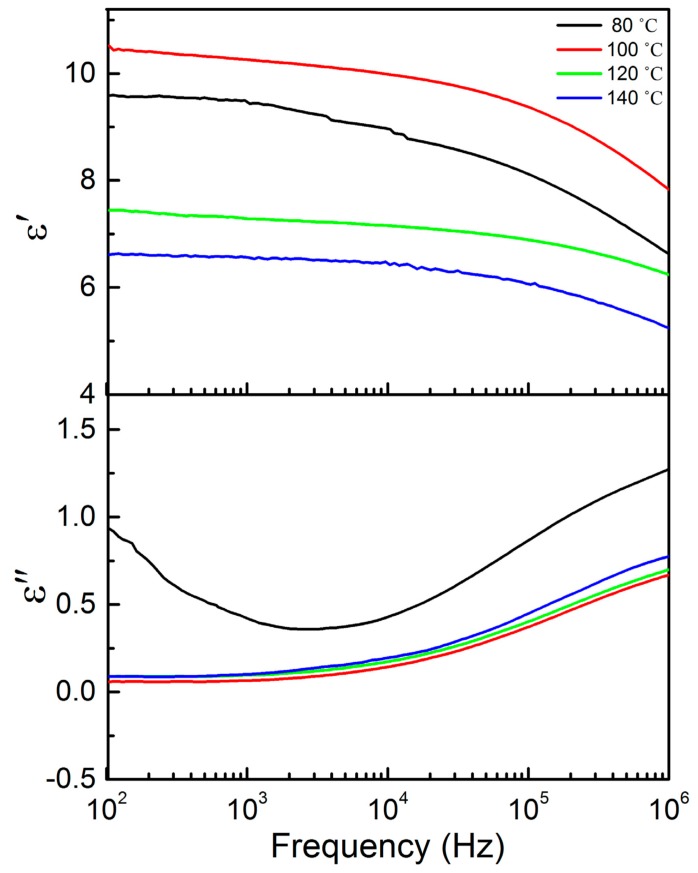
Room temperature dielectric frequency spectra of P(VDF-TrFE) treated with various annealing temperatures.

Figure 7 shows the frequency dependence of dielectric permittivity ε′ and dielectric loss ε″ for P(VDF-TrFE) annealed at 100 °C and measured from 30 °C to 140 °C at a temperature step size of 10 °C. It is shown that the dielectric of 100 °C annealed thin film at 1 kHz and room temperature has the highest value around ε′ = 10.5, and the loss was ε″ = 0.1. The annealing temperature applied to the thin films (100 °C) led to an increase in the dielectric constant. The enhancement of the crystalline structure of the thin film has resulted in the molecules aligning in a much ordered conformation with high molecular chain packing. As a consequence, the dipole density in the system increased and led to an increase in the dielectric constant. We observed the there is a jump of the dielectric constant from 90 °C to 100 °C, where the ferroelectric to paraelectric phase transition takes place. In the paraelectric phase (90 °C to 140 °C), the dielectric and relaxation strength continuously increase, and the relaxation frequency increases with increasing temperature. If the temperature is varied from 140 °C to 160 °C, the copolymer will experience phase changes from the paraelectric to the molten phase. It is important to note here that only one significant ε″ peak is observed at the MHz frequency (room temperature), which is attributed to both the crystalline and the noncrystalline regions [9]. In contrast, PVDF shows two relaxations near 1 Hz and 1 MHz, which are associated with crystalline and noncrystalline motion, respectively. Figure 8 illustrate the room temperature dielectric frequency spectra of P(VDF-TrFE) treated with various annealing temperature. We compared the room temperature complex dielectric spectra of copolymer thin films annealed at different temperatures. When the annealing temperature increased from 80 °C to 100 °C, dielectric increased from 9 to 10.5. However, the dielectric constant starts to decrease dramatically to 7.2 and 6.3, when the copolymer thin films are annealed at 120 °C and 140 °C, respectively. The XRD result indicates that this may be due to the decrease in the crystalline structure of the thin film. It can be noted that when the thin film was annealed at 80 °C, the dielectric constant at room temperature was high ε′ = 9, but the loss was the highest, ε″ = 0.5. This may be due to the poor crystallinity and porosity in the thin film, since at the annealing temperature (80 °C), the thermal energy provided is not enough for the growth of the crystalline structure and crystalline lamellae, as was shown in X-ray and FESEM.

### 3.6. Pyroelectric Analysis

The pyroelectric coefficient of P(VDF-TrFE) copolymer thin films was measured using the quasi-static pyroelectric measurement method. The static (zero-frequency) pyroelectric coefficient, *p*, was obtained by measuring the induced pyroelectric current *I_p_* during the heating of the sample from 26 to 28 °C at a nearly constant heating rate $\frac{dT}{dt}$ (2–3 °C/s) with an effective area of 1 mm^2^ for all samples. The relationship of the pyroelectric coefficient is given by [21–24].

$$p=\frac{I_{p}}{A\left(\frac{dT}{dt}\right)}$$

**Figure 9.**
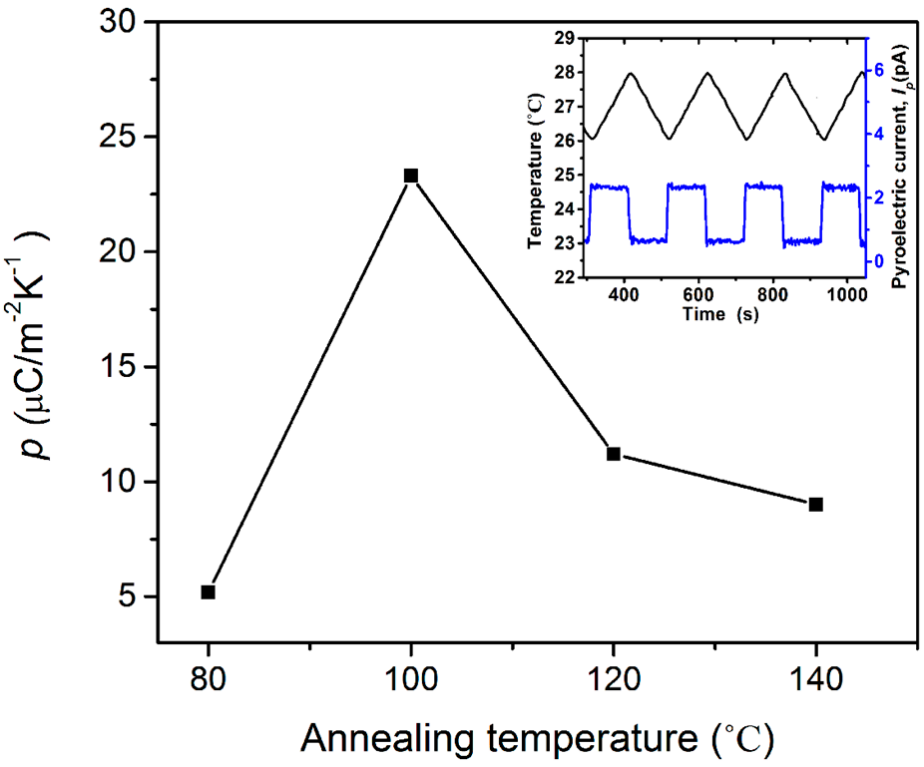
Pyroelectric coefficient, *p*, of P(VDF-TrFE) thin films with varying annealing temperature (T).

A rectangular waveform of pyroelectric current was obtained when a triangular temperature waveform was applied to the P(VDF-TrFE) thin films, with different annealing temperatures, as shown in the inset of Figure 9, which depicts the pyroelectric coefficients of the thin film annealed at four different annealing temperatures. It was found that the temperature of 100 °C is the optimum annealing temperature, with a pyroelectric coefficient of 24 μC/m^2^K. A poor pyroelectric coefficient has been obtained from the thin film annealed at 80 °C due to the low degree of the crystalline structure produced at that annealing temperature, as revealed by the XRD results. The pyroelectric coefficient has decreased considerably after annealing the thin film at 120 °C and 140 °C. This observation is related to the significant change in the crystalline structure of the thin films. The pyroelectric coefficients for the thin film annealed at these two annealing temperatures (120 and 140 °C) were reduced to 10 μC/m^2^K and 8 μC/m^2^K, respectively.

Combining X-ray, SEM, ferroelectric and pyroelectric results, we summarize the annealing induced structural changes in the PVDF-TrFE thin films as follows. The degree of the crystalline structure is one of the most important factors that affects *P*_r_ and *p*. This is mainly because its ferroelectricity and pyroelectricity are originated from the crystalline region. The molecule chains (*c*-axis) should be oriented parallel to the film surface, because polarization is induced in the *b*-axis direction normal to the chain axis. The overall polarization in the VDF copolymer could be improved by annealing in the paraelectric phase, in which active thermal motion allows chain molecules to rearrange their position and forms a highly crystalline phase, as shown in Figure 3. Figures 6 and 9, which show the dependence of *P*_r_ and *p* on the annealing temperature, are fairly consistent with the crystalline structure changes. This implies that values of *P*_r_ and *p* are governed by the degree of the crystalline structure. The large rod-like crystal grain, as shown in Figure 3b, is highly crystalline with the molecular chain aligned parallel to the substrate and uses the highest *P*_r_ = 94 mC/m^2^ and *p* = 24 μC/m^2^K. Copolymer annealed with such a simple method exhibited exceptional functional properties if compared to that of copolymers treated with a higher annealing temperature in the oven [20,25–27]. A further increase in annealing temperature will induce the elongation of the crystal into a needle-like structure, but decrease in the crystalline structure and its polarization.

## 4. Conclusions

The effect of annealing temperature on the crystalline structure of P(VDF-TrFE) (70/30) thin films (220 nm) has been investigated with a wide range of treated temperature (80–140 °C) below and above the Curie temperature to optimize the crystalline structure. The annealing temperature is an essential parameter that can be used to enhance the crystalline structure of the thin films and the related electrical properties (pyro-, ferro- and dielectric). From the obtained results, we found that the optimized crystalline structure was achieved at around 100 °C when the thin films were annealed with a hot plate. The thin films show very sharp and high intensity XRD peaks with a dominant *β* phase. The P(VDF-TrFE) thin film annealed at 100 °C exhibits a square-like hysteresis loop with high *P*_r_ = 94 mC/m^2^ and *E*_c_ = 80 MV/m. If the thin films were annealed with an oven, it required a higher optimum annealing temperature, which is about 140 °C. This annealing temperature is much closer to the melting point of the material and, thus, can cause many defects in the sample. The degree of crystalline structure decreases above the annealing temperature of 100 °C, as indicated by X-ray. The lamella length increases to 15 μm at 140 °C with cracks appearing on the surface of the thin film. The thin films also had poor ferroelectric and pyroelectric properties with high dielectric loss when annealed below the Curie temperature. Thus, the technique of annealing the thin film at 100 °C with a hot plate can be used to achieve P(VDF-TrFE) thin film with improved ferroelectric, dielectric and pyroelectric properties, which is suitable for many applications, such as energy storage, sensors and actuators.
